# Significance of dopamine D_1_ receptor signalling for steroidogenic differentiation of human induced pluripotent stem cells

**DOI:** 10.1038/s41598-017-15485-4

**Published:** 2017-11-09

**Authors:** Koji Matsuo, Masakatsu Sone, Kyoko Honda-Kohmo, Takafumi Toyohara, Takuhiro Sonoyama, Daisuke Taura, Katsutoshi Kojima, Yorihide Fukuda, Youichi Ohno, Mayumi Inoue, Akira Ohta, Kenji Osafune, Kazuwa Nakao, Nobuya Inagaki

**Affiliations:** 1 Department of Diabetes, Endocrinology and Nutrition, Kyoto University Graduate School of Medicine, 54 Shogoin Kawahara-cho, Sakyo-ku, Kyoto, 606-8507 Japan; 2 Center for iPS Cell Research and Application, Kyoto University, 53 Shogoin Kawahara-cho, Sakyo-ku, Kyoto, 606-8507 Japan; 3 Medical Innovation Center, Kyoto University Graduate School of Medicine, 53 Shogoin Kawahara-cho, Sakyo-ku, Kyoto, 606-8507 Japan

## Abstract

Human induced pluripotent stem cells (hiPSCs) are expected to be both a revolutionary cell source for regenerative medicine and a powerful tool to investigate the molecular mechanisms underlying human cell development *in vitro*. In the present study, we tried to elucidate the steroidogenic differentiation processes using hiPSC-derived intermediate mesoderm (IM) that is known to be the origin of the human adrenal cortex and gonads. We first performed chemical screening to identify small molecules that induce steroidogenic differentiation of IM cells expressing Odd-skipped related 1 (OSR1), an early IM marker. We identified cabergoline as an inducer of 3β-hydroxysteroid dehydrogenase, an essential enzyme for adrenogonadal steroidogenesis. Although cabergoline is a potent dopamine D_2_ receptor agonist, additional experiments showed that cabergoline exerted effects as a low-affinity agonist of D_1_ receptors by increasing intracellular cyclic AMP. Further analysis of OSR1^+^ cells transfected with steroidogenic factor-1/adrenal 4 binding protein revealed that D_1_ receptor agonist upregulated expression of various steroidogenic enzymes and increased secretion of steroid hormones synergistically with adrenocorticotropic hormone. These results suggest the importance of dopamine D_1_ receptor signalling in steroidogenic differentiation, which contributes to effective induction of steroidogenic cells from hiPSCs.

## Introduction

Adrenal insufficiency occurs when the adrenal cortex fails to produce sufficient levels of steroid hormones, which are essential for our survival. One type of insufficiency, glucocorticoid deficiency, presents a life-threatening condition requiring immediate treatment. Although glucocorticoid replacement therapy is a standard strategy to treat this condition, patients require treatment for their entire lives and, thus, are always at risk from its many side effects including adrenal crisis, obesity, osteoporosis, hypertension and glucose intolerance^1–5^.

Regenerative medicine using human pluripotent stem cells (hPSCs) is a new therapeutic option that is expected to be a potential solution to these problems^6–8^. Among classical endocrine organs, the differentiation of pancreatic β-cells from hPSCs has been the most thoroughly investigated^9,10^. Meanwhile, some reports describe the induction of various stem cell populations into steroid-producing cells. In 1997 Crawford *et al*. first reported the induction of mouse embryonic stem cells into steroidogenic cells using forced expression of steroidogenic factor-1/adrenal 4 binding protein (SF-1/Ad4BP), known as a transcriptional master regulator of steroidogenic genes^11^. However, the steroidogenic capacity was very limited because progesterone was the only steroid hormone produced in the presence of an exogenous substrate, 20α-hydroxycholesterol. More recently several groups have reported that both mouse and human mesenchymal stem cells (MSC) can be induced to differentiate into steroid-producing cells through forced expression of SF-1 and that the resultant steroid-producing cells produce a wider variety of steroid hormones^12–16^, but the MSC-derived steroid-producing cells have not been well characterised because there is no evidence that the steroid-producing cells naturally develop from the MSC. In 2012 we first reported the induction of both human embryonic stem cell (hESC)- and human induced pluripotent stem cell (hiPSC)-derived mesodermal cells into steroid-producing cells^17^. The forced expression of SF-1 and subsequent treatment with 8-bromoadenosine 3′,5′-cyclic monophosphate induced the mesodermal cells into steroidogenic cell lineage capable of secreting cortisol. Yet, the pathway by which hPSCs differentiate into steroidogenic cells has not been fully clarified.

The adrenal cortex, gonads and kidneys are thought to be derived from an identical origin, intermediate mesoderm (IM), during foetal development^12,18^. The adrenal cortex is derived from a thickening of IM, known as the gonadal ridge, at 4 to 5 weeks of gestation. Adrenogonadal progenitor cells divide into two distinct organs under the regulation of many transcriptional factors, including SF-1. Among markers specific for IM, Odd-skipped related 1 (OSR1) is known as one of the earliest to appear. Lineage-tracing experiments have indicated metanephric kidneys, gonads and adrenal glands are all derived from OSR1-positive (OSR1^+^) cells^19–21^. Recently, we established hiPSC lines containing green fluorescence protein knocked into OSR1 (OSR1-GFP)^22^. Using these lines, we have been able to sort IM cells from undifferentiated hiPSCs using flow cytometry.

Importantly, hPSCs are expected to not only be a cell source for regenerative medicine, but also a powerful tool to investigate the molecular mechanisms underlying human cell development *in vitro*. For this aim, chemical screening has reportedly been a useful method. Chen *et al*. developed a high-content screen to identify small molecules capable of increasing Pdx1-expressing pancreatic progenitors derived from hESCs^23^. Using a similar approach, Araoka *et al*. identified two compounds effective for differentiating hiPSCs/ESCs into IM cells^24^.

Therefore, in the present study, we used IM cells to perform a chemical screen for small molecules that increase steroidogenic enzyme expression. For primary screening, we determined that 3β-hydroxysteroid dehydrogenase type 2 (3β-HSD2) would be an appropriate marker, as it is an essential enzyme for adrenogonadal steroidogenesis expressed by IM cells. Our screen of approximately 3,500 compounds identified one small molecule, cabergoline, that upregulated 3β-HSD2 expression. Interestingly, further analysis revealed its mechanism of effect occurred via dopamine D_1_ receptors, not D_2_. We next analysed this effect in SF-1-transfected IM cells (thought to be more differentiated toward an adrenocortical fate) and observed that dopamine D_1_ receptor activation upregulated expression of 3β-HSD2 and other downstream steroidogenic enzymes, and increased steroid secretion. These results indicate that dopamine D_1_ receptor signalling has a role in steroidogenic differentiation.

## Results

### Chemical screen for small molecules that increase 3β-HSD-positivity in IM cells

**Figure 1:**
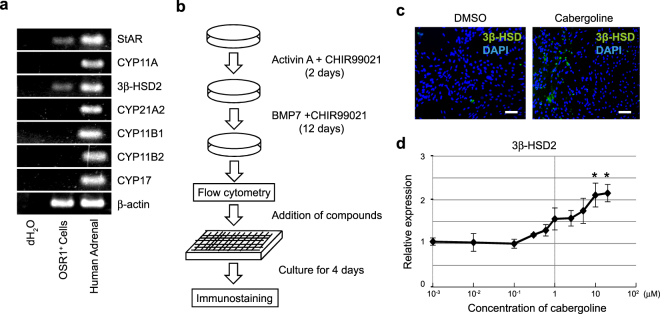
Chemical screening strategy and the effect of cabergoline. (**a)** RT-PCR analysis of steroidogenic enzyme mRNA expression in OSR1^+^ cells. **(b)** Schematic representation of induction of undifferentiated induced pluripotent stem cells into intermediate mesoderm and screening strategy used. **(c)** Effect of cabergoline on OSR1^+^ cells treated with 1 μM cabergoline for 4 days and then stained with an anti-3β-HSD antibody and DAPI. Scale bar, 50 μm. **(d)** Dose-dependent 3β-HSD2 mRNA expression curve in cabergoline-treated OSR1^+^ cells. Levels of 3β-HSD2 mRNA expression are shown as relative values compared with those of DMSO-treated controls. Expression levels are normalised to that of a housekeeping gene, β-actin. Student’s *t*-test was used for comparison between the experimental and control group. *P < 0.05. Data represent mean ± SEM of four independent experiments (n = 4).

The screen was designed to identify small molecules that promote differentiation of hiPSC-derived intermediate mesoderm cells toward steroidogenic cells. For this aim, we used a hiPSC reporter line (3D45) containing an OSR1 gene with GFP knocked-in (OSR1-GFP). First, to determine an appropriate marker indicating the induction of IM cells into steroidogenic cells, we examined the mRNA expression of steroidogenic enzymes in OSR1^+^ cells. Reverse transcription polymerase chain reaction (RT-PCR) analysis indicated mRNA expression of steroidogenic acute regulatory protein (StAR) and 3β-HSD2, but not other steroidogenic enzymes, such as cytochrome P450 (CYP) 11A1, CYP21A2, CYP11B1, CYP11B2 and CYP17, in IM cells (Fig. 1a). As 3β-HSD2 is an essential enzyme for adrenogonadal steroidogenesis, we determined 3β-HSD immunostaining would be an appropriate method for primary screening according to the strategy shown in Fig. 1b.

To collect starting material for this screen, IM cells were induced via embryoid body (EB) formation, as previously described^22^. Isolated OSR1-GFP^+^ cells were seeded onto 384-well plates at a density of 8,000 cells per well, and individual compounds were added to each well on the following day. Four days later, cells were stained with an anti-3β-HSD antibody. Wells in which the percentage of 3β-HSD-positive (3β-HSD^+^) cells was more than 10 times higher than cells treated with vehicle (1% DMSO) were considered as primary hits. The results of our primary screen of 3,648 compounds yielded 8 hits. We subsequently ruled out compounds exhibiting autofluorescence and identified one compound, cabergoline. The percentage of 3β-HSD^+^ cells in cabergoline-treated cultures (1.59%) was higher than that of DMSO-treated controls (0.07 ± 0.01%, Fig. 1c). Quantitative real-time PCR (qRT-PCR) analysis also showed increased 3β-HSD2 mRNA expression occurred in a dose-dependent manner for the concentrations examined (1 nM to 20 μM). Significant changes compared with DMSO-treated controls were observed between 10 and 20 μM (Fig. 1d). We determined that 20 μM of cabergoline would be the most appropriate concentration for further analysis because the dose showed the highest efficiency without cytotoxicity.

### Cabergoline upregulates 3β-HSD2 mRNA expression via activation of dopamine D_1_ receptors

**Figure 2:**
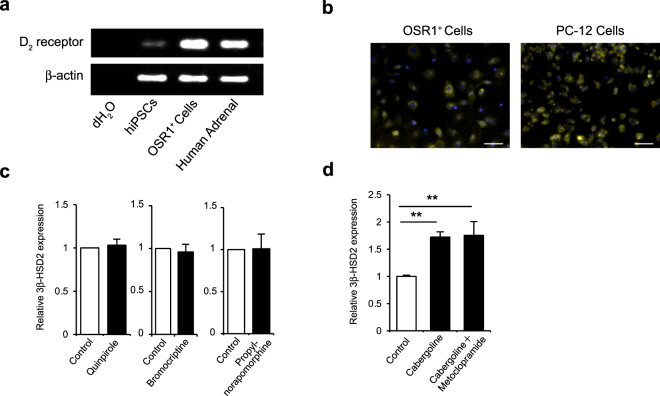
Expression of dopamine D_2_ receptors on OSR1^+^ cells and effects of D_2_ receptor agonists and an antagonist. (**a)** RT-PCR analysis of dopamine D_2_ receptor mRNA expression in OSR1^+^ cells. **(b)** Immunofluorescence staining of dopamine D_2_ receptors on OSR1^+^ cells. Rat adrenal chromaffin cell line PC-12 was also stained as a positive control because it is known to express dopamine D_1_ and D_2_ receptors^38,39^. Scale bar, 50 μm. **(c)** Levels of 3β-HSD2 mRNA expression in OSR1^+^ cells treated with dopamine D_2_ receptor agonists, including 10 μM quinpirole, 1 μM bromocriptine and 10 μM propylnorapomorphine. Expression levels are normalised to that of a housekeeping gene, β-actin. Student’s *t*-test was used for comparison of two groups. *P < 0.05. Data represent mean SEM of four independent experiments (n = 4). **(d)** Levels of 3β-HSD2 mRNA expression in OSR1^+^ cells treated with cabergoline and a dopamine D_2_ receptor antagonist. Cells were treated with 20 µM cabergoline under daily administration of 10 μM metoclopramide. Statistical analysis was performed using two-tailed unpaired Student’s *t*-test with Holm–Bonferroni correction. **P < 0.01. Data represent mean ± SEM of four independent experiments (n = 4).

Cabergoline is known as a potent, high-affinity, long-acting agonist of dopamine D_2_ receptors, one of two subclasses of dopamine receptors. Therefore, we initially assumed that D_2_ receptor signalling upregulated 3β-HSD2 mRNA expression in OSR1^+^ cells. First, we examined D_2_ receptor expression in OSR1^+^ cells using RT-PCR analysis and found mRNA expression levels in OSR1^+^ cells were equivalent to human adrenal glands (Fig. 2a). To confirm their expression at the protein level, we next performed immunofluorescence staining of D_2_ receptors on OSR1^+^ cells (Fig. 2b). We then investigated the effects of other D_2_ receptor agonists by treating OSR1^+^ cells with three types of D_2_ receptor agonists, 10 μM quinpirole, 1 μM bromocriptine and 10 μM propylnorapomorphine, for 4 days. However, none of these agonists promoted steroidogenic differentiation of OSR1^+^ cells (Fig. 2c). Moreover, the promotive effect of cabergoline was not affected by daily administration of 10 μM metoclopramide, a D_2_ receptor antagonist (Fig. 2d). These results suggest enhancement of steroidogenic differentiation by cabergoline was not elicited by its D_2_ receptor agonist activity. Supporting this notion, cabergoline only improved differentiation at relatively high concentrations in spite of its extremely high affinity for D_2_ receptors. Therefore, we next presumed the activity of this compound resulted from its function as a low-affinity agonist of D_1_ receptors.

**Figure 3:**
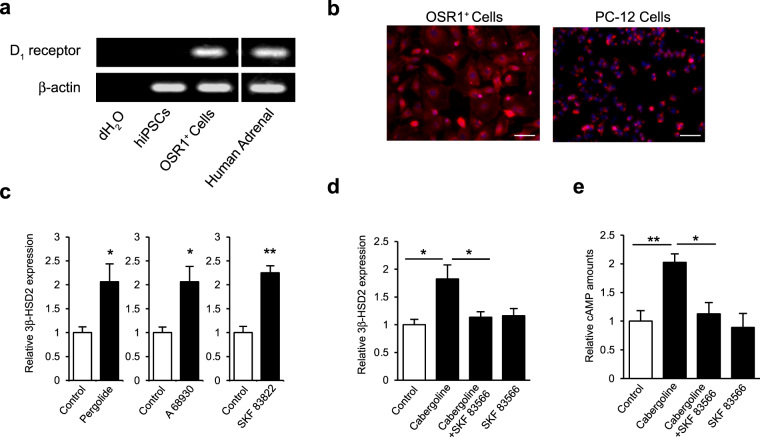
Expression of dopamine D_1_ receptors on OSR1^+^ cells and effects of D_1_ receptor agonists and an antagonist. (**a)** RT-PCR analysis of dopamine D_1_ receptor mRNA expression in OSR1^+^ cells. **(b)** Immuno-fluorescence staining of dopamine D_1_ receptors on OSR1^+^ cells. PC-12 cells are shown for comparison (positive control). Scale bar, 50 μm. **(c)** Levels of 3β-HSD2 mRNA expression in OSR1^+^ cells treated with dopamine D_1_ receptor agonists. Cells were treated with 10 μM pergolide, 10 μM A 68930 and 1 μM SKF 83822. Expression levels are normalised to levels of a housekeeping gene, β-actin. Student’s *t*-test was used for comparison of two groups. *P < 0.05; **P < 0.01. Data represent mean ± SEM of four independent experiments (n = 4). **(d)** Levels of 3β-HSD2 mRNA expression in OSR1^+^ cells treated with cabergoline and D_1_ receptor antagonist. Cells were treated with 20 µM cabergoline and 10 μM SKF 83566. Statistical analysis was performed by one-way ANOVA followed by the Tukey-Kramer test. *P < 0.05. Data represent mean ± SEM of four independent experiments (n = 4). **(e)** Levels of intracellular cAMP in OSR1^+^ cells treated with 20 µM cabergoline and 10 μM SKF 83566. Statistical analysis was performed by one-way ANOVA followed by the Tukey-Kramer test. *P < 0.05; **P < 0.01. Data represent mean ± SEM of six independent experiments (n = 6).

To test this hypothesis, we examined dopamine D_1_ receptor expression in OSR1^+^ cells. First, RT-PCR analysis showed D_1_ receptors were not expressed in undifferentiated hiPSCs, but were in OSR1^+^ IM cells (Fig. 3a). In addition, we confirmed expression of D_1_ receptors on OSR1^+^ cells by immunofluorescence staining (Fig. 3b). Next, OSR1^+^ cells treated for 4 days with three types of D_1_ receptor agonists (10 µM pergolide, 10 µM A 68930 or 1 µM SKF 83822) all upregulated 3β-HSD2 mRNA expression (Fig. 3c). In addition, qRT-PCR analysis showed treatment of OSR1^+^ cells with a D_1_ receptor antagonist (10 μM SKF 83566) blocked the promotive effect of simultaneous cabergoline (20 μM) administration (Fig. 3d), suggesting that cabergoline enhances 3β-HSD2 mRNA expression via activation of dopamine D_1_ receptors. To confirm cabergoline exerts its effects via D_1_ receptors, we then assayed intracellular cyclic AMP (cAMP), which is known as a second messenger of D_1_ receptors, after treatment with cabergoline and SKF 83566. While cabergoline significantly increased intracellular cAMP, simultaneous administration of SKF 83566 cancelled the cabergoline-mediated increase in cAMP (Fig. 3e). Taken together, these results suggest that dopamine D_1_ receptor signalling enhances steroidogenic differentiation by increasing intracellular cAMP.

### Addition of cabergoline or a D_1_ receptor agonist does not induce expression of other downstream steroidogenic enzymes in OSR-1^+^ IM cells

**Figure 4:**
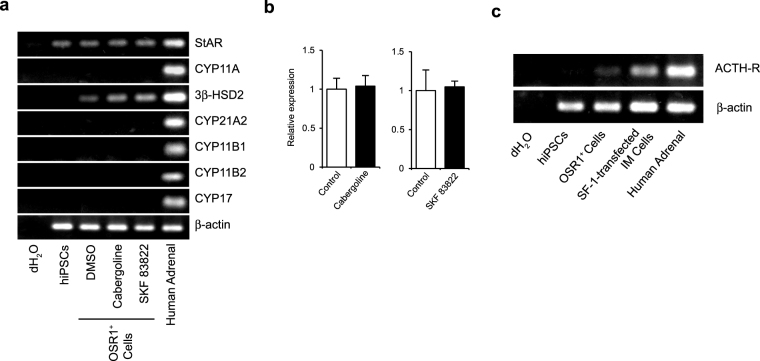
Effect of cabergoline and a dopamine D_1_ receptor agonist on steroidogenic enzyme and ACTH-R expression. (**a)** RT-PCR analysis of steroidogenic enzyme mRNA expression. **(b)** StAR mRNA expression levels in OSR1^+^ cells treated with 20 μM cabergoline and 1 µM SKF 83822. Expression levels are normalised to levels of a housekeeping gene, β-actin. Student’s *t*-test was used for comparison of two groups. *P < 0.05. Data represent mean ± SEM of four independent experiments (n = 4). **(c)** RT-PCR analysis of mRNA expression of ACTH receptor in OSR1^+^ cells and SF-1-transfected OSR1^+^ IM cells.

Additional experiments examining the effects of cabergoline and SKF 83822 on mRNA expression of other steroidogenic enzymes failed to detect any expression, except for StAR and 3β-HSD2 (Fig. 4a). Additional qRT-PCR analysis showed mRNA expression of StAR was not changed by the addition of either of these drugs (Fig. 4b). Upon examining adrenocorticotropic hormone (ACTH) receptor mRNA expression in OSR1^+^ cells, we found it was hardly detectable (Fig. 4c). Furthermore, ACTH stimulation did not affect steroidogenic gene mRNA expression (Supplementary Fig. S1). In addition, SF-1 mRNA expression was undetectable by RT-PCR analysis (Supplementary Fig. S2).

### SF-1 transfection of OSR1^+^ IM cells

Dopamine D_1_ receptor agonists did not enhance mRNA expression levels of steroidogenic enzymes other than 3β-HSD2 in OSR1^+^ cells, a result we believe may stem from generally low expression of steroidogenic enzymes in these cells. Recently we have reported that forced expression of SF-1, known to be a master regulator of steroidogenic gene transcription, induces hESC- and hiPSC-derived mesodermal cells into steroid-producing cells. Therefore, we transfected SF-1 into OSR1^+^ cells and examined the effect of a dopamine D_1_ receptor agonist.

OSR1^+^ IM cells were induced as described in the Methods section. After 1–3 days in culture, isolated OSR1^+^ cells were transfected with plasmid DNA encoding SF-1, with a transfection efficacy of approximately 60% (Supplementary Fig. S3). Expression levels of mRNAs for various steroidogenic enzymes were elevated in SF-1-transfected IM cells (Supplementary Fig. S4). Moreover, RT-PCR analysis showed the emersion of ACTH receptors (Fig. 4c).

### Dopamine D_1_ receptor agonist upregulates expression of mRNA for various steroidogenic enzymes in SF-1-transfected IM cells

**Figure 5:**
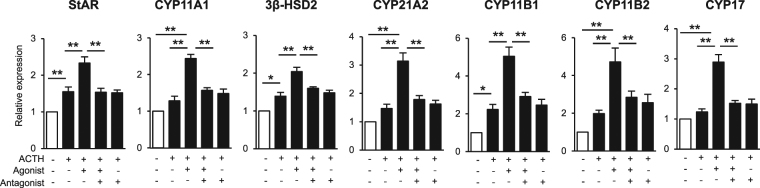
Effect of ACTH and a dopamine D_1_ receptor agonist on steroidogenic enzyme mRNA expression. Steroidogenic enzyme mRNA expression levels in SF-1-transfected OSR1^+^ cells treated with 2.4 µM ACTH, 1 µM SKF 83822 and 10 µM SKF 83566. Expression levels are normalised to levels of a housekeeping gene, β-actin. Statistical analysis was performed by one-way ANOVA followed by the Tukey-Kramer test. *P < 0.05; **P < 0.01. Data represent mean ± SEM of eight independent experiments (n = 8).

Based on these results, we examined the effect of SKF 83822, a D_1_ receptor agonist, in the presence of ACTH stimulation. SF-1-transfected OSR1^+^ cells were treated for 24 h with combinations of 1 μM SKF 83822, 2.4 μM ACTH and 10 μM SKF 83566. qRT-PCR analysis showed that SKF 83822 upregulated the mRNA expression levels of StAR, CYP11A1, CYP21A2, CYP11B1, CYP11B2, CYP17 and 3β-HSD2, and these effects were synergistically promoted by ACTH stimulation (Fig. 5). Moreover, the promotive effects of SKF 83822 were completely blocked by SKF 83566, a D_1_ receptor antagonist. Cells treated with ACTH alone showed significantly upregulated expression of StAR, 3β-HSD2 and CYP11B1. Upon measuring steroid hormones secreted into culture medium during two days of culture, we found that the cells produced cortisol, aldosterone and dehydroepiandrosterone (DHEA). Secretion of cortisol was significantly increased in response to both SKF 83822 and ACTH (Table 1). Cells treated with a combination of SKF 83822 and ACTH showed significant increases in the production of aldosterone and DHEA.

**Table 1:** Levels of steroid hormones secreted into culture medium.

	Control	ACTH	ACTH + SKF 83822
Cortisol (pg/ml)	415 ± 19.9	**560 ± 26.2	* ^**†**^1136 ± 215
Aldosterone (pg/ml)	14.5 ± 0.9	19.5 ± 2.0	*27.1 ± 3.5
DHEA (pg/ml)	2139 ± 278	2291 ± 274	*3059 ± 126

Culture medium was collected for steroid hormone measurements after a 48 h of incubation with 2.4 µM ACTH and/or 1 µM SKF 83822. Statistical analysis was performed using two-tailed unpaired Student’s *t*-test with Holm-Bonferroni correction. *, *vs Control P* < 0.05; **, *vs Contol P* < 0.01; ^†^, *vs ACTH P* < 0.05. Data represent mean ± SEM of five independent experiments (n = 5).

## Discussion

Using a chemical screening method, the present study identified a compound, cabergoline, that increases 3β-HSD expression in OSR1^+^ IM cells. Additional experiments revealed this effect was exerted through dopamine D_1_ receptor activation. Upon examining the significance of this receptor in steroidogenic cells induced by forced expression of SF-1 in OSR1^+^ IM cells, we found that a dopamine D_1_ receptor agonist upregulates various steroidogenic enzymes and increases the production of steroid hormones.

From an embryological point of view, it is known that sympathoadrenal progenitors from the neural crest infiltrate into the adrenal gland at 7 to 8 weeks of gestation and differentiate into catecholamine-producing adrenal medulla cells^25^. Catecholamines in the foetus are known to be present in chromaffin tissues by 10 to 15 weeks of gestation, with concentrations increasing closer to birth. Considering the development of chromaffin cells *in utero*, intermediate mesoderm (OSR1^+^ cells), an early embryonic germ layer present at 4 to 5 weeks of gestation, seemed to be too immature to fully examine the effects of dopamine receptor agonists. Mimicking the signals during embryonic steroidogenic differentiation to generate more mature steroid-producing cells should allow the effects of a dopamine D_1_ receptor agonist to be examined to a greater extent. Therefore, we transfected SF-1 into OSR1^+^ IM cells and differentiated them toward steroidogenic cells.

In our chemical screening, immunofluorescence staining with the anti-3β-HSD antibody showed that the percentage of 3β-HSD^+^ cells in cabergoline-treated cultures was 1.59%. Additional experiments revealed this promotive effect was exerted via dopamine D_1_ receptors. Furthermore, strong and homogenous expression of dopamine D_1_ receptors on OSR1^+^ IM cells was confirmed by immunofluorescence staining. Based on these results, we believe that cabergoline has an effect on the steroidogenic enzyme in all cabergoline-treated OSR1^+^ cells, but OSR1^+^ IM cells are heterogeneous populations in terms of their steroidogenic capacity and likelihood to differentiate into the steroidogenic cell lineage.

To our knowledge, this is the first report describing the significance of dopamine D_1_ receptors in human developing steroidogenic cells. Dopamine receptors are divided into two subclasses based on their biochemical and pharmacological characteristics: D_1_-like and D_2_-like receptors. Their functions are associated with regulation of cAMP and protein kinase A (PKA) through G protein-mediated signalling. In general, dopamine D_1_-like receptors are coupled with Gsα to stimulate production of the second messenger cAMP and PKA activity. In contrast, D_2_-like receptors negatively regulate cAMP production and PKA activity^26^.

In previous studies, the focus was on D_2_ receptors in the adrenal cortex, rather than D_1_ receptors. D_2_ receptors are known to be expressed in normal adrenal cortex. They are strongly expressed in the zona glomerulosa and reticularis, but hardly expressed in the zona fasciculata^27,28^. Conversely, D_1_ receptors are reportedly expressed in normal adrenal gland (presumably in the zona fasciculata) and some cortisol-secreting adenomas, but not in aldosterone-secreting adenomas. While several reports have suggested D_2_ receptors negatively regulate aldosterone production, the effects of dopamine and dopamine agonists on adrenocortical steroidogenesis remains controversial^28–32^. Pivonello *et al*. demonstrated biphasic effects of cabergoline on steroid production by human adrenal hyperplasia-derived cells. In their report, cabergoline increased aldosterone secretion at a low dose and decreased its secretion at a high dose. They suggested that the biphasic effects of dopamine agonists are related to the adverse effects of D_1_-like and D_2_-like receptors, although they did not clarify the distribution of D_1_ receptors in the zona glomerulosa. In this study, we found that cabergoline increased 3β-HSD2 expression in OSR1^+^ cells, and the promotive effect at the high concentration was mediated by D_1_ receptors. However, no suppressive effect was seen at the low concentration. We propose two reasons for the difference in these effects of the dopamine agonists. First, the difference may be related to the distribution of each dopamine receptor subclass in these cells. In OSR1^+^ cells, immunofluorescence staining with anti-dopamine receptor antibodies indicated strong expression of dopamine D_1_ receptors compared with D_2_ receptors. Therefore, the predominance of D_1_ receptors resulted in the absence of suppressive effects on the steroidogenic enzymes by caberoline and other D_2_ receptor agonists in our experiments. Second, the difference may stem from the difference in cell characters between premature and mature steroidogenic cells. To investigate this possibility, we carried out additional experiments examining the effect of a D_1_ receptor agonist on steroidogenic enzymes in adult adrenocortical cells using human adrenocarcinoma cells (H295R) treated with 1 μM SKF 83822 for 4 days, as previously described. However, qRT-PCR analysis did not show a promotive effect on steroidogenic enzyme mRNA expression (Supplementary Fig. S5). Although these results may reflect low dopamine D_1_ receptor expression compared with human adrenal (Supplementary Fig. S6), they may also indicate dopamine D_1_ receptor signalling is important during the developmental stage of adrenocortical cells, but not in adult steroidogenesis. Indeed, our results indicate that dopamine D_1_ receptor signalling promotes expression of steroidogenic enzymes in premature steroidogenic cells.

In this study, steroidogenic cells induced by forced expression of SF-1 produced cortisol, aldosterone and DHEA. Foetal DHEA synthesis is elevated because of low 3β-HSD2 expression and high CYP17 expression during early gestation^33^. Therefore, relatively high concentrations of DHEA observed in the present study confirmed that these experiments mimicked the physiological differentiation processes of human steroidogenic cells.

In 2012, we first reported that forced expression of SF-1 induces hiPSCs-derived mesodermal cells into steroidogenic cells^17^. To evaluate the efficacy of the differentiation method described in the present study, mRNA expression levels of steroidogenic enzymes in steroid-producing cells induced by the present method were compared with the preserved samples induced by our previous method. As a result, qRT-PCR analysis revealed that mRNA expression levels of various steroidogenic enzymes except for CYP21A2 were significantly higher in cells induced in the present study (Supplementary Fig. S7). This result suggests that the present method with the dopamine D_1_ receptor agonist is more effective for induction of steroid-producing cells. The absence of significant superiority in mRNA expression of CYP21A2 indicates the possibility that induction of CYP21A2 needs a longer culture duration after SF-1 transfection. In addition, steroidogenic cells differentiated in the present study expressed ACTH receptors and showed responsiveness to ACTH stimulation, which had not been shown in our previous report. This result supports the notion that OSR1^+^ cells isolated in the present study are a more specific source for the steroidogenic cell lineage, because OSR1 is a marker for intermediate mesoderm that differentiates into the adrenal cortex and gonads. Consequently, we propose that the method in the present study is more appropriate for induction of steroid-producing cells. Therefore, we conclude that the present study contributes to the progress of methods for induction of steroid-producing cells from hiPSCs.

The human adrenal cortex is responsible for inducing differentiation and catecholamine production in the adrenal medulla. Glucocorticoids provided by adrenocortical cells reportedly enable sympathoadrenal progenitors to differentiate into chromaffin cells^34^. Moreover, glucocorticoids induce phenylethanolamine N-methyltransferase, an indispensable enzyme for methylation of norepinephrine to form epinephrine. Furthermore, chromaffin cells have been shown to be necessary to maintain adrenocortical differentiation and functions by experiments using the frog or bovine adrenal cortex^35–37^. Our results may suggest that adrenal medulla-cortical interactions are also necessary for human adrenocortical differentiation.

In summary, our study demonstrates that dopamine D_1_ receptor activation upregulates mRNA expression of steroidogenic enzymes in IM-derived adrenogonadal progenitor cells. Although further investigation is required for complete elucidation of the differentiation mechanisms underlying human adrenogonadal formation, these results suggest the importance of dopamine D_1_ receptor signalling in steroidogenic differentiation, which contributes to effective induction of steroidogenic cells from hiPSCs.

## Methods

### Cell culture and differentiation

Human iPSCs (3D45, OSR1-GFP knock-in) were cultured on feeder layers of mitomycin C-treated mouse embryonic fibroblasts in Primate ES medium (ReproCELL, Yokohama, Japan) containing 1% penicillin/streptomycin (Sigma-Aldrich, St. Louis, MO) and 4 ng/ml recombinant human basic fibroblast growth factor (Peprotech, Rocky Hill, NJ). Cells were passaged every 7 days using phosphate-buffered saline (PBS) containing 0.25% trypsin (Thermo Fisher Scientific, Waltham, MA), 1 mg/ml collagenase IV (Thermo Fisher), 20% KnockOut™ Serum Replacement (KSR; Thermo Fisher Scientific) and 1 mM CaCl_2_.

OSR1^+^ cells were induced via EB formation as previously described^22^. In brief, undifferentiated hiPSCs were collected by dissociation using the solution previously described for passaging and then rinsed with Stage 1 medium [DMEM/F12 + Glutamax (Thermo Fisher) containing 0.5% penicillin/streptomycin and 2% foetal bovine serum (FBS; HyClone™; GE Healthcare Life Sciences, Marlborough, MA)]. Cells were replaced onto 10-cm dishes containing Stage 1 medium supplemented with 100 ng/ml recombinant human/mouse/rat activin A (R&D Systems, Minneapolis, MN) and 2 μM CHIR99021 (Axon Medchem BV, Groningen, the Netherlands). EBs were seeded onto gelatine-coated plates on the third day of culture and maintained for an additional 12 days in Stage 2 medium (DMEM/F12 + Glutamax containing 0.1 mM Non-Essential Amino Acid Solution, 0.5% penicillin/streptomycin, 0.55 mM 2-mercaptoethanol, 10% KSR), 100 ng/ml recombinant human bone morphogenetic protein 7 (ProSpec Bio, East Brunswick, NJ) and 2 μM CHIR99021.

H295R cells (ATCC^®^ CRL-2128; American Type Culture Collection, Manassas, VA) were cultured in DMEM containing 1% penicillin/streptomycin (Sigma-Aldrich), 2 mM L-glutamine (Sigma-Aldrich) and 10% FBS (Thermo Fisher Scientific). Cells were passaged every 7 days using 0.25% trypsin.

### Flow cytometry

Cells were detached using Accumax® (Innovative Cell Technologies, San Diego, CA), collected, and then washed with Hank’s Balanced Salt Solution (Thermo Fisher Scientific). Dead cells ware stained with BD Via-Probe™ (BD Biosciences, San Jose, CA). Cells were sorted using a fluorescence-activated cell sorter (FACS) Aria II (BD Biosciences), according to the manufacturer’s instructions. OSR1-GFP^+^ cells were collected in Stage 2 medium containing 10 μM Y-27632 (Wako, Tokyo, Japan).

### Chemical screening

Chemical screening was performed using a chemical library provided by JST Yamanaka iPS Cell Special Project, which was delivered in 384-well racks. Sorted OSR1^+^ cells were seeded onto gelatin-coated 384-well plates at a density of 8,000 cells per well and incubated overnight. The following day, individual compounds (1 µM final concentration) were added to each well in modified Stage 2 medium consisting of DMEM/F12 + Glutamax, 0.1 mM Non-Essential Amino Acid Solution, 0.5% penicillin/streptomycin, 0.55 mM 2-mercaptoethanol and 5% KSR. Four days later, cells were stained with an anti-3β-HSD antibody and the number of 3β-HSD^+^ cells was automatically analysed by an IN Cell Analyzer (GE Healthcare Life Sciences). Data were assessed as fold changes compared with the 1% DMSO control. Wells in which the percentage of 3β-HSD^+^ cells was more than 10 times higher than control wells treated with 1% DMSO were considered as primary hits.

### Immunostaining

Cells were fixed in either PBS containing 4% paraformaldehyde for 10 min at room temperature or 70% ethanol for 30 min at 4 °C. After washing with PBS, cells were incubated overnight at 4 °C with a mouse monoclonal anti-human 3β-HSD antibody (37-2) (1:200; Santa Cruz Biotechnology, Santa Cruz, CA), rabbit polyclonal anti-dopamine D_1_ receptor antibody (1:500; abcam, Cambridge, UK) and mouse monoclonal anti-D_2_ receptor antibody (1:50; Santa Cruz Biotechnology). In the chemical screening, Polyclonal Rabbit Anti-Mouse Immunoglobulins/FITC (1:200; Dako, Santa Clara, CA) were used as the secondary antibody. For immunostaining of dopamine receptors, Goat Polyclonal Anti-Mouse IgG (Alexa Flour 555, 1:500; abcam) and Donkey Anti-Rabbit Polyclonal IgG (Alexa Flour 647, 1:500; abcam) were used as secondary antibodies. They were incubated for 1 h at room temperature, and then nuclei were visualised with DAPI.

### Chemicals

Cabergoline, metoclopramide, bromocriptine, propylnorapomorphine, quinpirole, pergolide and A68930 were purchased from Sigma-Aldrich. SKF 83822 and SKF 83566 were purchased from Tocris (Bristol, UK).

### DNA transfection

Approximately 5 × 10^5^ cells were transfected with 2 μg of expression plasmid encoding SF-1 driven by the cytomegalovirus promoter (pCMFlag-hsNR5A1; Riken BRC, Saitama, Japan). Transfection was performed with Nucleofector™ (Lonza, Basel, Switzerland), an electroporation-based method for transferring nucleic acids into cells, according to the manufacturer’s instructions. Transfection efficiency was approximately 60% (Supplementary Fig. S3).

### RT-PCR and quantitative real-time PCR (qRT-PCR)

Total RNA was extracted using either an RNeasy® Mini Kit (Qiagen, Venlo, the Netherlands) or NucleoSpin® RNA Kit (Takara Bio, Kusatsu, Japan), according to the manufacturers’ instructions. cDNA was synthesised from 1 μg of total RNA using a PrimeScript™ RT Reagent Kit (Takara Bio); the reaction was carried out at 37 °C for 15 min and terminated by heating at 85 °C for 5 s. For RT-PCR, the cDNA samples were subjected to PCR amplification using a thermal cycler. PCR was performed using Takara Taq™ (Takara Bio) according to the manufacturer’s instructions with the following protocol: initial denaturation at 94 °C for 2 min; followed by 25–40 cycles of 94 °C for 30 s, 60 °C for 30 s, 72 °C for 30 s, and extension at 72 °C for 10 min. Ten microlitres of resulting PCR products were electrophoresed on a 1.5% agarose gel and visualised by ethidium bromide staining. Quantitative real-time PCR was performed using an ABI PRISM 7300 Sequence Detection System (Applied Biosystems, Carlsbad, CA) and SYBR® Premix Ex Taq II Kit (Takara Bio). PCR protocol was as follows: denaturation at 95 °C for 10 s, followed by 40 cycles of 95 °C for 5 s and 60 °C for 31 s. Primers used in this study are listed in Table S1. Levels of mRNA expression were normalised to those of a housekeeping gene, β-actin.

### Measurement of intracellular cAMP

OSR1^+^ cells were incubated on 24-well plates with 20 µM cabergoline and 10 μM SKF 83566 for one hour and lysed with 0.1 M HCl. Levels of intracellular cAMP were analysed using Direct cAMP ELISA kit (Enzo Life Sciences, Farmingdale, NY) following the manufacturer’s instructions.

### Measurement of steroid hormones in culture medium

After overnight incubation, SF-1-transfected steroidogenic cells were treated with 1 μM SKF 83822 and/or 2.4 μM ACTH (Peptide Institute, Osaka, Japan). After 48 h of incubation, culture medium was collected for steroid hormone measurements using commercially available enzyme immunoassay kits for DHEA (Enzo Life Sciences), aldosterone (Cayman Chemicals, Ann Arbor, MI) and cortisol (Arbor Assays, Ann Arbor, MI).

### Statistical analysis

All data are expressed as mean ± standard error of the mean (SEM). For comparison of two or three groups, statistical analysis was performed using a two-tailed unpaired Student’s *t*-test with Holm-Bonferroni correction. For comparisons of more than three groups, statistical analysis was performed by one-way analysis of variance (ANOVA) followed by the Tukey-Kramer test. Data were considered significant (P < 0.05) or highly significant (P < 0.01).

## Electronic supplementary material

supplementary information
