# Supplementary material for: Significance of dopamine D1 receptor signalling for steroidogenic differentiation of human induced pluripotent stem cells
Source: Sci Rep. 2017 Nov 9;7:15120. doi: 10.1038/s41598-017-15485-4 (PMC5680317; doi:10.1038/s41598-017-15485-4)
Supplement: Supplementary file 1 — supplementary information [file 41598_2017_15485_MOESM1_ESM.pdf]

Supplementary Information

Significance of dopamine D_1 receptor signalling for steroidogenic differentiation of human induced pluripotent stem cells

Koji Matsuo, Masakatsu Sone*, Kyoko Honda-Kohmo, Takafumi Toyohara, Takuhiro Sonoyama, Daisuke Taura, Katsutoshi Kojima, Yorihide Fukuda, Youichi Ohno, Mayumi Inoue, Akira Ohta, Kenji Osafune, Kazuwa Nakao, Nobuya Inagaki

*To whom correspondence should be addressed.

Email: sonemasa@kuhp.kyoto-u.ac.jp

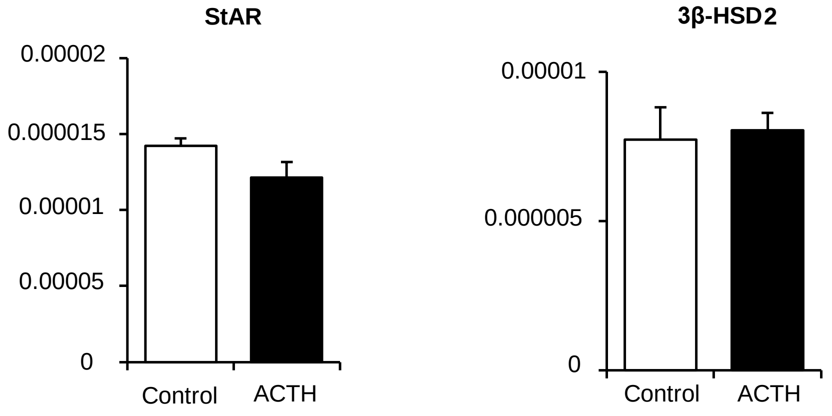

Supplementary Figure S1. qRT-PCR analysis of steroidogenic enzyme mRNA in OSR1$^+$ cells treated with or without ACTH stimulation. Steroidogenic enzyme mRNA expression levels in OSR1$^+$ cells treated with or without 2.4 μM ACTH for 24 h. Steroidogenic enzymes other than StAR and 3β-HSD2 were not detected. Expression levels are normalised to levels of a housekeeping gene, β-actin. Data represent mean ± SEM of three independent experiments (n = 3).

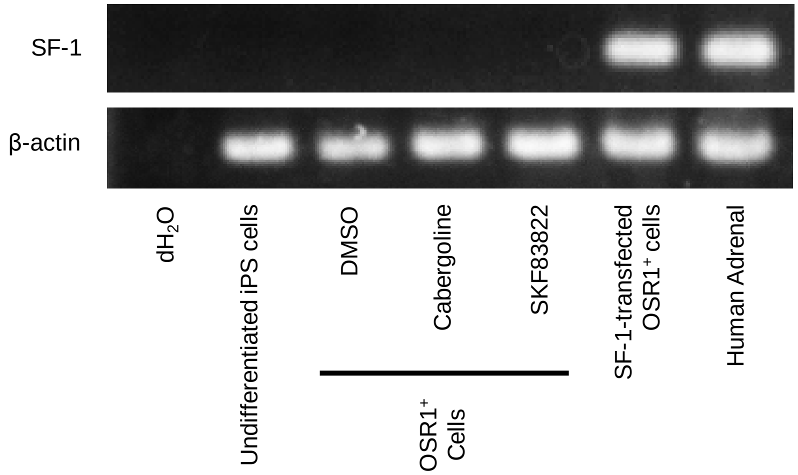

Supplementary Figure S2. Expression of steroidogenic factor-1 (SF-1). OSR1^{+} cells were treated with 1 µM DMSO, 20 µM cabergoline, or 1 µM SKF83822 for four days. SF-1-transfected OSR1^{+} cells were harvested on the day after DNA transfection.

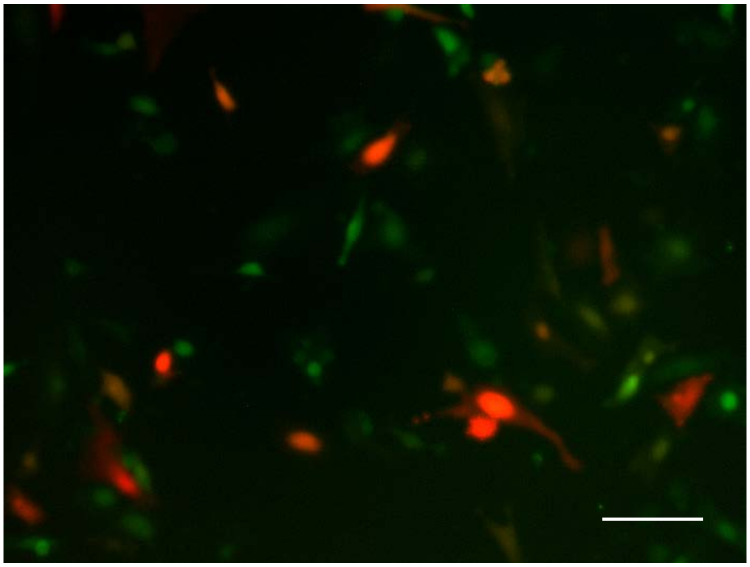

Supplementary Figure S3. Transfection of mCherry-C1 vector into OSR1$^+$ cells. DNA transfection efficacy using Nucleofector (LONZA) was calculated as the transfection efficiency of mCherry-C1 vector into OSR1$^+$ cells by the same protocol as that used for pCMFlag-hsNR5A1. Scale bar = 100 μm.

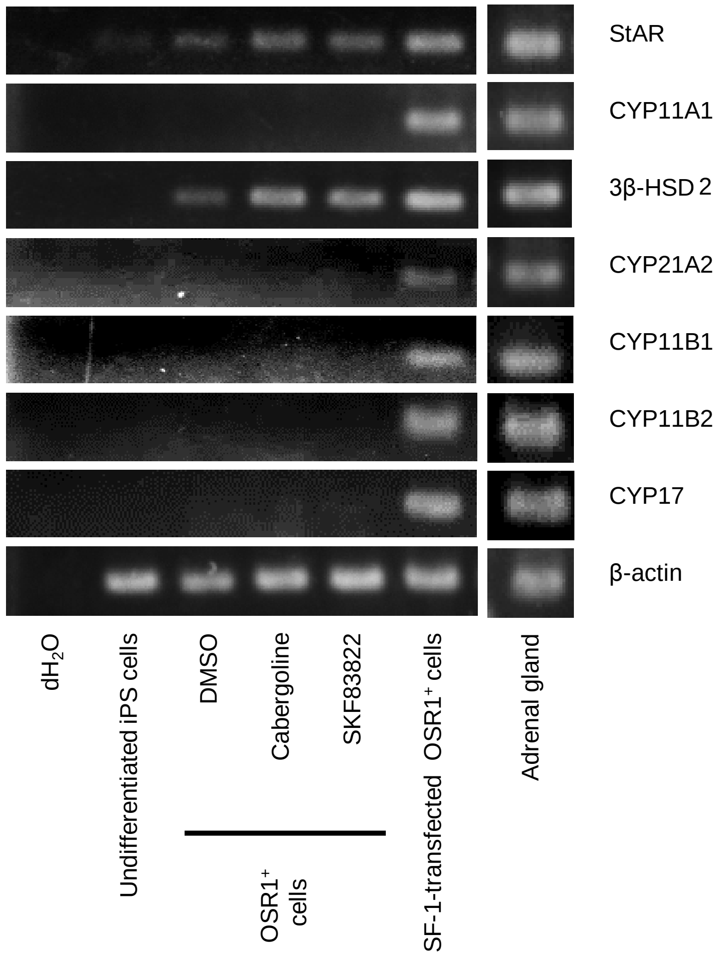

Supplementary Figure S4. Expression of steroidogenic enzymes. $OSR1^+$ cells were treated with 1 μM DMSO, 20 μM cabergoline, or 1 μM SKF83822 for four days. SF-1-transfected $OSR1^+$ cells were harvested on the day after DNA transfection. Results obtained with human adrenal gland are shown for comparison.

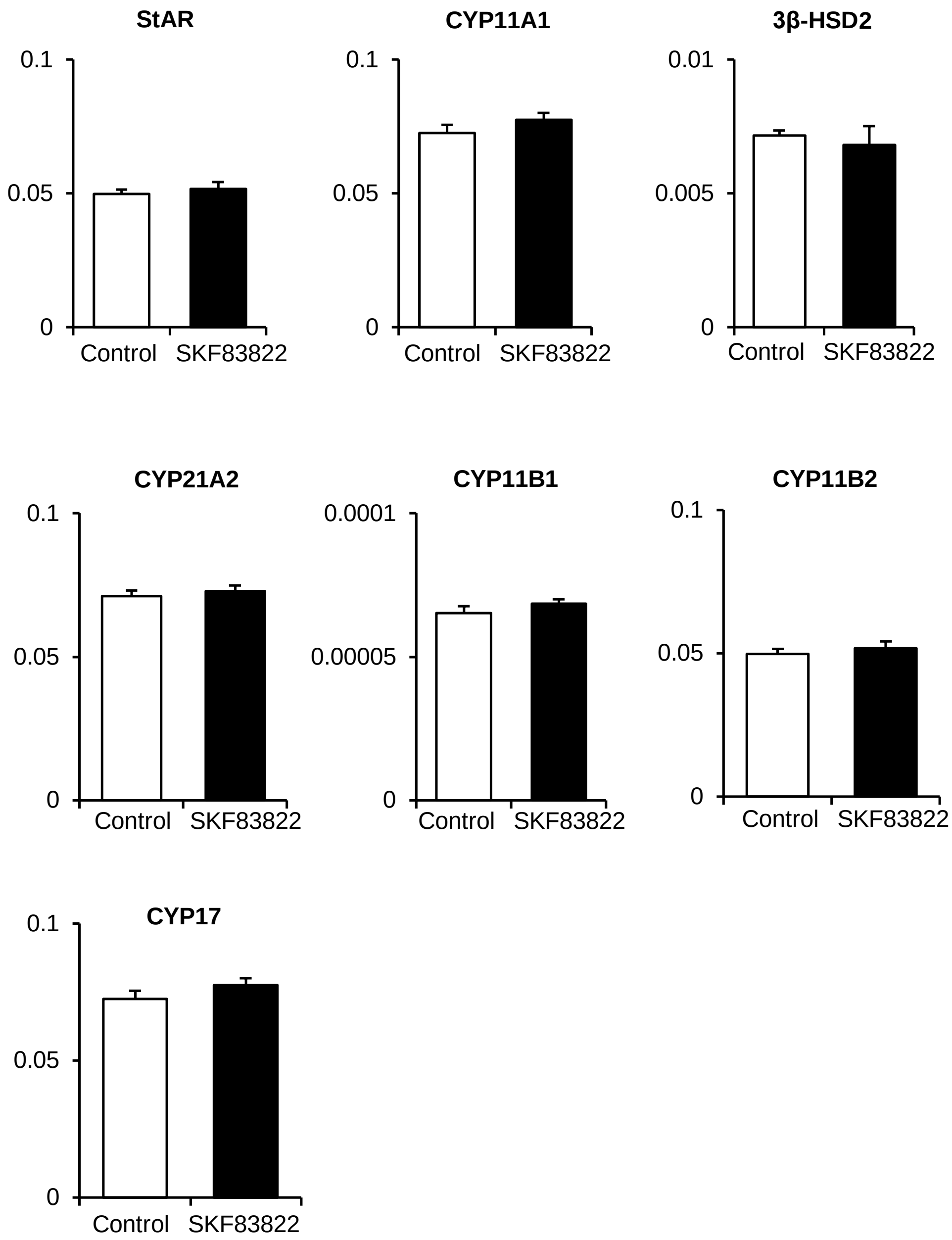

Supplementary Figure S5. Effect of dopamine D_1 receptor agonist on human adrenocarcinoma cells (H295R). H295R cells were treated with 1 μM DMSO (control) or 1 μM SKF 83822 for four days. Expression levels are normalised to levels of a housekeeping gene, β-actin. Data represent mean ± SEM of four independent experiments (n = 4).

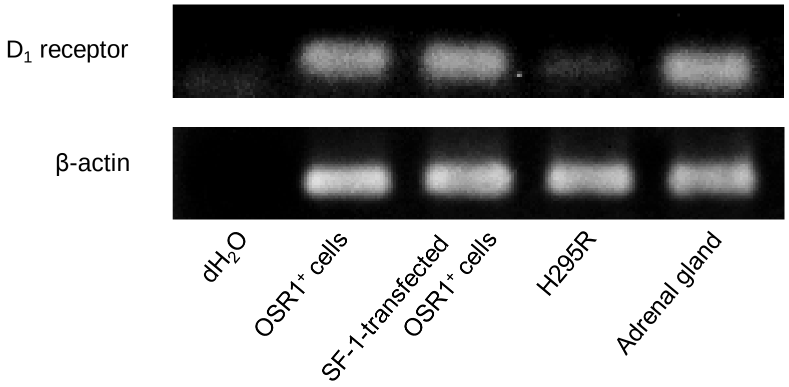

Supplementary Figure S6. Expression of dopamine D_1 receptors on human adrenocarcinoma cells (H295R). RT-PCR analysis of mRNA expression of dopamine D_1 receptors in H295R. Results obtained with $OSR1^+$ cells, SF-1-transfected $OSR1^+$ cells and human adrenal gland are shown for comparison.

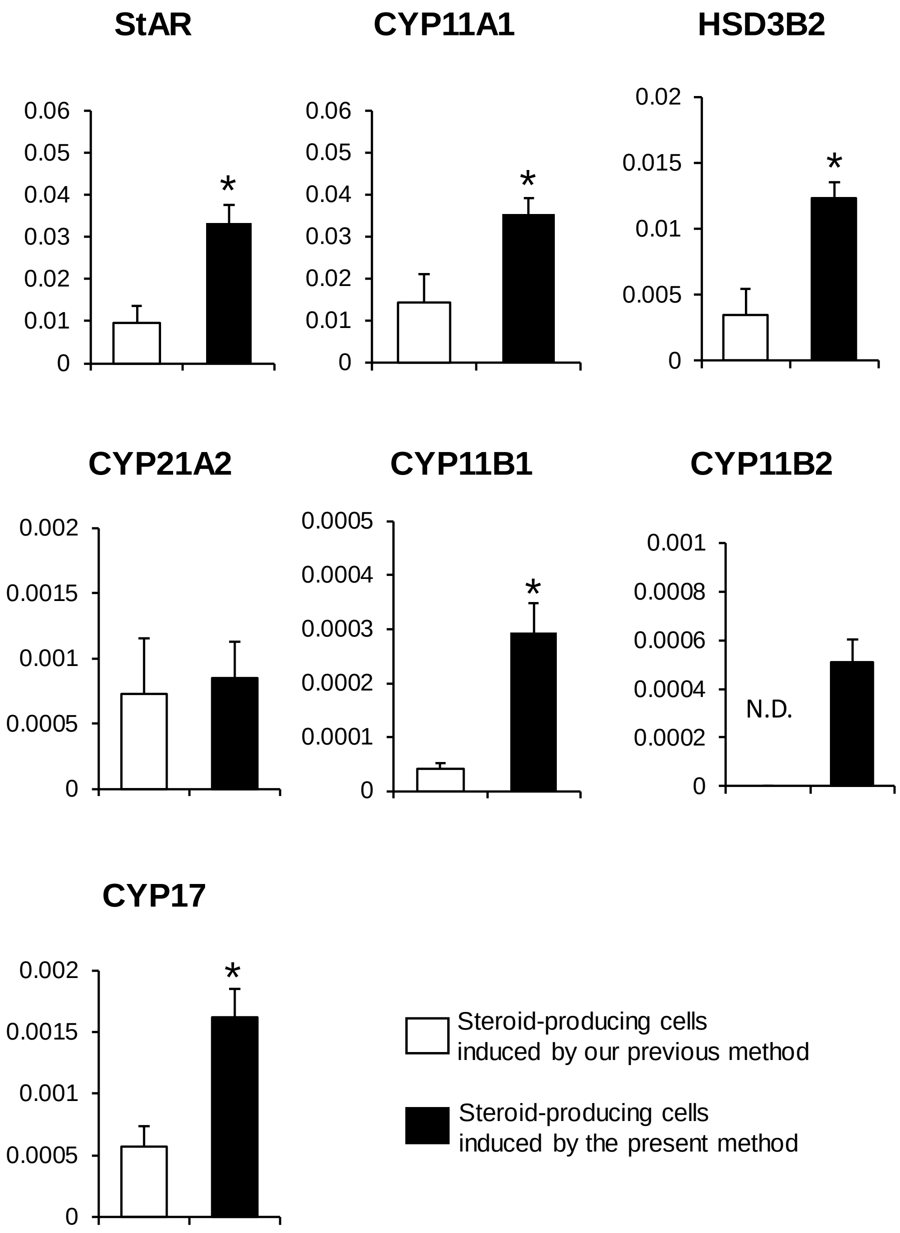

Supplementary Figure S7. Comparison between the steroid-producing cells induced by the present method and those induced by our previous method. mRNA expression levels of steroidogenic enzymes in steroid-producing cells induced by the present method and those induced by our previous method described in 2012 are shown. The cDNAs of the steroid-producing cells induced by our previous method were synthesised from the preserved RNA samples and mRNA expression levels in both groups were analysed in the same PCR runs. Expression levels are normalised to levels of a housekeeping gene, β-actin. Student's *t*-test was used for comparison of two groups. $*P < 0.05$. Data represent mean ± SEM of six independent experiments (n = 6). N.D., Not detectable.

Supplementary Table S1. Primer sequences.

Primer name	Sequence
STAR Forward	TGGGCCCTTATGTACCCACCTA
STAR Reverse	CAGCGCATGGCATTCTTGA
CYP11A1 Forward	TGACAATGGCTGGCTAAACC
CYP11A1 Reverse	CGATGACATAAACCGACTCCAC
HSD3B2 Forward	TGGACAAGGCCTTCAGACCAG
HSD3B2 Reverse	AATGATACAGGCGGTGTGGATG
CYP21A2 Forward	AAAGTGGGCAGACTTTGCTG
CYP21A2 Reverse	TGAGCTTCTTGTGGGCTTTC
CYP17 Forward	GCGATCAGAAGCTGGAGAAG
CYP17 Reverse	CGCCACGAAGACAGGAAAG
CYP11B1 Forward	TCCTGTTGAATGCGGAACTG
CYP11B1 Reverse	TCATCAGCAAGGGGAAACACC
CYP11B2 Forward	TAGAAGCCATCAAGGCCAAC
CYP11B2 Reverse	CAAAGAGCGTCATCAGCAAG
ACTB Forward	TGGCACCCAGCACAATGAA
ACTB Reverse	CTAAGTCATAGTCCGCCTAGAAGCA
NR5A1 Forward	ATGGCCGACCAGACCTTCA
NR5A1 Reverse	TAGATGTGGTCGAACACCAGCAG
DRD1 Forward	CGAATAATGCCATAGAGACGGTGA
DRD1 Reverse	CTTGGAGATGGAGCCTCGTG
DRD2 Forward	CACCACCAACTACCTGATCGTC
DRD2 Reverse	CAGTGAATCCTGCTGAATTTCCA
MC2R Forward	TGATGCTGCAATGCTCTGTGA
MC2R Reverse	CAAAGCTGTTGACAAAGGCAGAA

Primers used in this study are listed above.
